# Effects of Perioperative Oral Nutrition Supplementation in Malaysian Patients Undergoing Elective Surgery for Breast and Colorectal Cancers—A Randomised Controlled Trial

**DOI:** 10.3390/nu14030615

**Published:** 2022-01-30

**Authors:** Ting Xuan Wong, Wei Xiang Wong, Seong Ting Chen, Shu Hwa Ong, Sangeetha Shyam, Nurzarina Ahmed, Khairul Hazim Hamdan, Raflis Ruzairee Awang, Mohd Razali Ibrahim, Kandasami Palayan, Winnie Siew Swee Chee

**Affiliations:** 1 Division of Nutrition & Dietetics, School of Health Sciences, International Medical University, Bukit Jalil, Kuala Lumpur 57000, Malaysia; wong.tingxuan@student.imu.edu.my (T.X.W.); wong.weixiang@student.imu.edu.my (W.X.W.); chenseongting@imu.edu.my (S.T.C.); ongshuhwa@imu.edu.my (S.H.O.); sangeethashyam@imu.edu.my (S.S.); 2 Department of General Surgery, Hospital Tuanku Ja’afar, Seremban 70300, Malaysia; z8rin@yahoo.com; 3 Department of General Surgery, Hospital Kuala Lumpur, Kuala Lumpur 50586, Malaysia; k_hazim@yahoo.com (K.H.H.); drraflis@gmail.com (R.R.A.); mdrazali73@yahoo.com (M.R.I.); 4 Department of Surgery, School of Medicine, International Medical University, Bukit Jalil, Kuala Lumpur 57000, Malaysia; pkan3033@gmail.com

**Keywords:** perioperative nutrition care, breast cancer, colorectal cancer, non-severe malnutrition, surgical outcomes

## Abstract

This study aimed to investigate the effectiveness of preoperative and an extended 90-days postoperative use of ONS among patients undergoing elective surgery for breast and colorectal cancers. Ninety-one patients were randomised into (i) Group SS received ONS up to 14 days preoperatively and postoperatively up to discharge, (ii) Group SS-E received ONS up to 14 days preoperatively, postoperatively up to discharge and for an extended 90-days after discharge and (iii) Group DS received ONS postoperatively up to discharge. Preoperatively, SS had significantly higher body weight (66.1 ± 15.3 kg vs. 62.5 ± 12.0 kg, *p* = 0.010) and BMI (26.8 ± 6.8 kg/m^2^ vs. 26.1 ± 6.7 kg/m^2^, *p* = 0.022) than DS when adjusted for baseline values. Postoperatively, SS-E had significantly higher handgrip strength (26 ± 9 kgF vs. 24 ± 6 kgF, *p* = 0.044) than DS at 90-days post-discharge after adjusted for preoperative values. At 90-days post-discharge, the proportions of patients in SS with albumin < 35 g/d, CAR ≥ 0.1, mPINI ≥ 0.4, mGPS score 1 or 2 were significantly reduced while in SS-E, the reduction in proportions of patients with high hsCRP and mPINI ≥ 0.4 was significant compared to upon discharge. Preoperative ONS had modest benefits in attenuating weight loss whilst postoperative supplementation up to 90-days post-discharge improved handgrip strength and inflammatory prognostic markers.

## 1. Introduction

Colorectal and breast cancers are among the most common cancers reported worldwide, contributing up to 9.4% and 6.9% of total cancer deaths, respectively [1]. About half of the total cancer incidence (49.3%) and mortality (58.3%) occur in Asian countries [1]. In developing countries such as Malaysia, the management of new cancer cases imposes substantial costs amounting to RM108 million (USD 26 million) per year, excluding the costs for therapies, surveillance, and palliative care [2].

Surgery is the mainstay of treatment for cancer [3] and malnutrition remains a concern among cancer patients undergoing surgery with curative intent [4]. Colorectal and breast cancers are among the less aggressive cancer types and patients often do not exhibit conventional criteria for malnutrition such as low serum albumin, BMI, or weight loss [5,6]. They are predominantly overweight or obese upon diagnosis [7,8], however, malnutrition can occur following the disease trajectory as the primary tumour itself could exert metabolic alterations in the body while the surgical stress further exacerbates the nutrition status of the patients [9,10,11].

In addition, cancer has complex interactions with inflammation and can trigger a cascade of the tumour-promoting inflammatory responses [12]. During the process, pro-inflammatory cytokines such as interleukin-6 (IL-6) are released from the immune system causing alteration to the liver synthesis of plasma proteins [9]. Albumin is one of the negative acute-phase proteins that decreases while C-reactive protein is one of the positive acute-phase proteins that increases in the blood circulation [9]. Accordingly, markers of systemic inflammation derived from C-reactive protein (CRP), albumin, neutrophils, and lymphocytes, such as C-reactive protein-to-albumin ratio (CAR), modified prognostic inflammatory and nutritional index (mPINI), neutrophil-to-lymphocyte ratio (NLR), and modified Glasgow prognostic score (mGPS) have been studied to indicate disease prognosis for cancers. These inflammatory prognostic markers have been demonstrated to correlate with malnutrition, postoperative complications, poor survival, and worse prognosis among patients with operable cancer [13,14,15]. Concurrently, surgical incision could also exacerbate the inflammation by directly causing tissue injury, driving the surgical stress response and development of sepsis [16].

Most guidelines recommend the provision of nutrition intervention among malnourished surgical patients to provide nutrients that support vital organ function while acute medical treatment is offered to preserve nutrient stores in the body for recovery [9]. Perioperative use of oral nutrition supplements (ONS) is also evidenced by systematic reviews as beneficial for attenuation of weight loss, improvement of nutrition intake without suppressing food intake or appetite, reduction of complications including infections and pressure ulcers, reduction of mortality and decreased length of hospital stay, as compared to the non-supplemented controls [17,18]. However, the benefits of ONS remain inconclusive among the non- and mildly malnourished patient population. More studies are also needed to ascertain the use of ONS in improving the clinicopathological outcomes such as inflammatory markers given the ongoing chronic inflammation in cancer.

The optimal duration of oral nutrition supplementation provision to the surgical cancer patients is also debated [19]. The average duration of preoperative supplementation to demonstrate clinical benefits is 15 days, ranging from 5 days to 4 weeks [6]. In addition, it is also ambiguous if the extension of supplementation after discharge would be beneficial in the recovery process. These data are essential in the management of malnutrition in cancer as they can directly impact the overall cost of treatment, especially to patients from developing countries.

Therefore, this study is aimed to address these research gaps by determining the effects of perioperative ONS supplementation on nutrition status and clinical outcomes among patients with breast and colorectal cancers undergoing elective surgery.

## 2. Materials and Methods

### 2.1. Study Design

This study was an open-label, multi-arm, parallel-group randomised controlled trial conducted at two tertiary hospitals in Negeri Sembilan and Kuala Lumpur, Malaysia. Ethical approval was obtained from the Malaysian Medical Research and Ethics Committee (NMRR-18-392-40035 (IIR)) and the International Medical University Joint Committee on Research and Ethics (IMU R 204/2017). The trial was registered on ClinicalTrial.gov (identifier: NCT04400552) and the full study protocol has been published previously [20].

### 2.2. Study Setting and Participants

The screening, recruitment, and baseline data collection were conducted at the surgical outpatient clinics of Hospital Tuanku Ja’afar, Negeri Sembilan and Hospital Kuala Lumpur, Malaysia. Data collection was performed at the surgical outpatient clinic and at the ward one day prior to discharge from the hospital. Subsequently, patients were followed up at the surgical outpatient clinic at 30-days and 90-days post-discharge. Potential patients were provided with a study information sheet detailing the objectives and the risks of the study. All patients provided written informed consent before screening for eligibility. Patients who met all the following criteria were recruited: male or female from all ethnicities, aged between 25 and 65 years, diagnosed with breast or colorectal cancer and scheduled for elective surgery, BMI not less than 18.0 kg/m^2^, stabilised comorbidities according to the A.S.A Physical Status Classification System Class 1 and 2 [21] and present with at least two characteristics of A.N.D/A.S.P.E.N diagnosis of adult malnutrition, i.e., insufficient energy intake, weight loss, loss of muscle mass, loss of subcutaneous fat, localised or generalised fluid accumulation and diminished functional status as measured by handgrip strength [22]. Patients who presented with any of the following criteria were excluded: on enteral or parenteral feeding, currently pregnant or lactating, on chemotherapy or radiotherapy, had undergone total gastrectomy or ileostomy, with advanced or metastasised cancer, terminal diseases, decompensated liver or renal disease, dementia or major concurrent metabolic problem such as uncontrolled diabetes, currently on steroid therapy and currently receiving treatment on Enhanced Recovery After Surgery (ERAS) protocol.

### 2.3. Randomisation and Blinding

Recruited patients were randomised into one of the three intervention arms in the ratio of 1:1:1 without stratification. The randomisation codes were generated using an open source software [23] by a research team member who was not involved in the data collection. The block randomisation method was employed and block sizes of either 3, 6, or 9 were used to minimise predictability. The allocation sequence was acquired by the graduate research assistant who conducted data collection and intervention assignment upon each successful recruitment to prevent selection bias. Both the patients and the research team members were not blinded to the intervention as this was designed as an open-labelled study.

### 2.4. Intervention Allocation

The three invention arms were named Group SS, Group SS-E, and Group DS. Group SS were prescribed with ONS for 5 to 14 days in addition to their usual diet preoperatively and postoperatively until being discharged from the hospital. Group SS-E were prescribed with ONS for 5 to 14 days in addition to their usual diet preoperatively, postoperatively until being discharged from the hospital, and for an extended period of 90-days post-discharge. Group DS received the usual care where the patients followed their usual diet preoperatively and only consumed ONS postoperatively until being discharged from the hospital.

The ONS was a standard milk-based formula, containing macronutrients and fortified with micronutrients (Appeton Wellness Recovery, Kotra Pharma (M) Sdn Bhd). Each recommended serving of ONS had 4 levelled scoops of powder (55 g) prepared with 210 mL of lukewarm water and patients were asked to consume 3 servings of ONS a day during the supplementation period. In total, the prescribed 3 servings of ONS provided an additional 750 kcal of energy, 33 g of protein, 83 g of carbohydrate (inclusive of 15 g of fiber) and 30 g of fat (inclusive of 17.4 g of monounsaturated fatty acid, 5.4 g of polyunsaturated fatty acid, 3.9 g of saturated fatty acid and 0.3 g of trans fatty acid) a day to the patients. The ONS was provided to patients in pre-packed cans. Compliance in the supplemented groups was determined by the percentage of actual amount consumed (g) from the prescribed amount (g) for consumption. Patients were provided a logbook to record the consumption of ONS and reminded fortnightly through phone calls or text messages to encourage compliance.

### 2.5. Data Collection and Outcome Measures

The primary outcomes were body weight, body mass index (BMI), and serum albumin level while secondary outcomes were handgrip strength (HGS), high-sensitivity C-reactive protein (hsCRP), and Interleukin-6 (IL-6). Data were collected at baseline, 1-day prior to surgery, upon discharge, 30-days, and 90-days post-discharge. Preoperative changes were defined as the change of the outcomes from baseline to 1-day prior to surgery. Changes from baseline to 30- and 90-days post-discharge were assessed for the effects of postoperative ONS.

Body weight (kg) was measured using a calibrated weighing scale (Tanita HD-325, Tanita Corporation, Tokyo, Japan) and height (m) was measured using a light-weight portable stadiometer (Seca 213, Seca, Hamburg, Germany). BMI was expressed as the weight (kg) divided by height (m^2^). Two measurements were made to compute the mean values.

Handgrip strength (kgF) was measured using an analogue hydraulic dynamometer (Jamar Hydraulic Hand Dynamometer 5030J1, Sammons Preston Rolyan, Bolingbrook, IL, USA) on the dominant hand. The measurement was repeated thrice with a 1-min interval to obtain the highest value.

A total of 8 mL of venous blood was drawn by a phlebotomist and centrifuged to aliquot the serum samples. The serum samples were stored at −80 degrees Celsius and analysed after collection of all 5 visits. Serum albumin and high-sensitivity C-reactive protein (hsCRP) were analysed using an automated sample analyser (Siemens Advia^®^ 1800 Clinical Chemistry System, Siemens Healthcare GmbH, Munich, Germany) and expressed in g/L and mg/L, respectively. Interleukin-6 (IL-6) was also analysed by an automated sample analyser (Siemens Immulite^®^ 2000 XPi immunoassay system, Siemens Healthcare GmbH, Munich, Germany) and expressed in pg/mL.

The C-reactive protein-related prognostic markers were C-reactive protein/albumin ratio (CAR) and Modified Prognostic Inflammatory and Nutritional Index (mPINI). CAR was computed based on the value of hsCRP level divided by albumin level. The cut-off value of ≥0.1 was adopted to indicate the poor prognosis of the treatment outcome [15]. The mPINI was derived from the CAR values and it stratified patients as having no risk (<0.4), low risk (0.4 to 1.2), moderate risk (1.2 to 2.0), or high risk (>2.0) of infectious and inflammatory complications [13]. The albumin-related prognostic marker was Modified Glasgow Prognostic Score (mGPS) and it is the combination of the albumin and CRP levels to predict survival among patients with operable cancer [14]. Patients with high CRP (>10 mg/L) and hypoalbuminemia (<35 g/L) were scored 2, patients with only high CRP (>10 mg/L) were scored 1, and patients with neither of the values were scored 0 [13]. The neutrophil-related prognostic marker used was the neutrophil–lymphocyte ratio (NLR). It was expressed as the ratio between the absolute neutrophil and lymphocyte counts. The values ≥5 were considered abnormal and associated with malnutrition and inflammatory response [13,14]. For all the inflammatory prognostic markers, higher values were associated with poorer prognosis.

### 2.6. Statistical Analysis

Statistical analyses were performed using the Statistical Package for the Social Science software (SPSS version 26, IBM, New York, NY, USA). The data were assessed for normality using Shapiro Wilk’s test and described based on the skewness of the data. Normally distributed data were expressed as mean ± standard deviation while skewed data were expressed as median with interquartile range. Categorical data were described in frequency and percentage. The analysis of the outcomes was conducted using the per-protocol and Intention-To-Treat (ITT) approaches. The per-protocol approach included patients who completed all study visits and Intention-To-Treat (ITT) approach imputed missing data using the last observation carried forward (LOCF) method. The primary analysis employed repeated measures ANOVA (RMANOVA) for the evaluation of time * group interactions for continuous variables. The secondary analysis was done by using the analysis of covariance (ANCOVA), controlling for baseline values. ANCOVA was restricted to a per-protocol basis. Pearson’s Chi-square test was used to detect differences in categorical variables between groups, and McNemar Chi-squared test was used to detect changes in categorical variables within groups. *p*-value < 0.05 was considered statistically significant for all tests.

### 2.7. Sample Size Calculation

The sample size was calculated to detect a post-supplementation between-group difference in serum albumin of at least 4 g/L. This has been shown to lead to a 25% reduction in morbidity among patients with normal serum albumin level of 38 g/L [24]. With an expected standard deviation of 6.5 g/L and a 5% alpha error at 80% power, a minimum of 23 patients was required per group. After accounting for the 30% drop-out rate, a total of 90 patients were required to be allocated into the three groups.

## 3. Results

### 3.1. Study Participants

**Figure 1:**
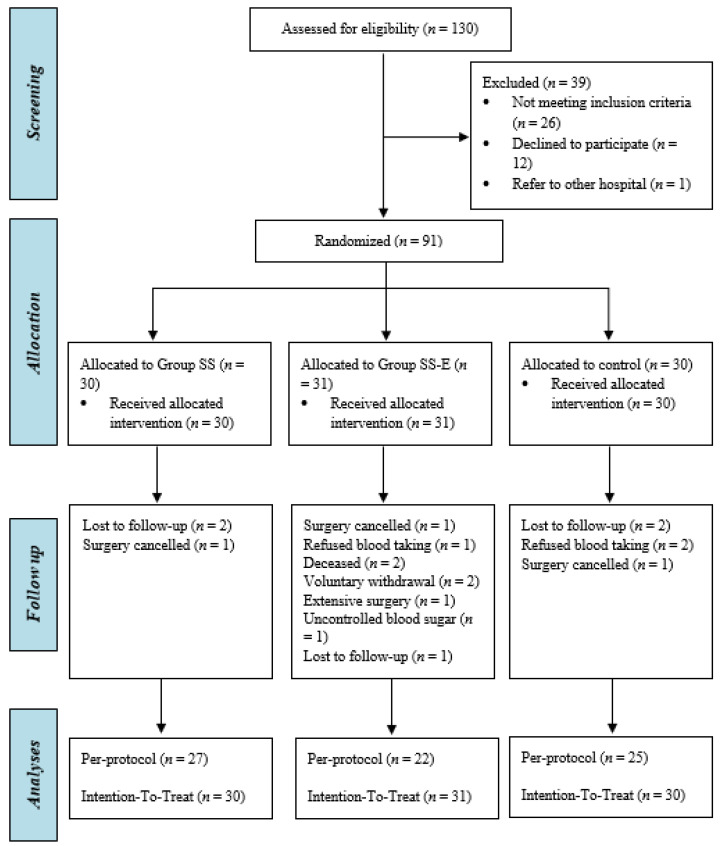
CONSORT flow diagram.

Figure 1 shows the trial enrolment in the form of a CONSORT flow diagram, in which 130 patients were approached from December 2018 to January 2021, and provided written consent to be assessed for eligibility to enter the study. Among them, 39 patients were ineligible for the trial as shown in Figure 1. The remaining 91 patients were enrolled in the study and randomized into group SS (*n* = 30), SS-E (*n* = 31), and DS (*n* = 30). Throughout the study period, a total of 17 patients (19%) dropped out of the study and the reasons are stated in Figure 1.

### 3.2. Baseline Characteristics

The distribution of age, sex, cancer types, comorbidities, body weight, BMI, nutritional status, HGS, albumin, hsCRP, and IL-6 across the intervention groups were comparable at baseline (Table 1). The type of surgery for colorectal cancers was mainly resection of tumour with partial resection of colon involved (95%) and mastectomy for breast cancers (60%).

### 3.3. Supplementation Duration and Compliance

Table 2 shows the supplementation duration and compliance rate of the patients. The preoperative supplementation duration was 8 (IQR: 6-14) days with an overall compliance rate of 85%. The duration of supplementation did not differ significantly between Group SS and SS-E (*p* = 0.278). All patients were supplemented for 4 (IQR: 3–5) days during hospitalisation with no significant difference between all groups (*p* = 0.539) and the compliance to supplementation during the post-surgery hospitalisation was 64%. The postoperative supplementation duration at 30-days post-discharge was 29 days (IQR: 26–35) with 77% compliance rate while the duration of supplementation was extended another 63 days (IQR: 56–68 days) at 75% compliance rate in Group SS-E. The common reasons for compliance rate below 80% were insufficient dosage of ONS consumed, reduced appetite, forgetfulness, and postoperative discomfort such as feeling dizzy, pain, or bloating. Nevertheless, none of the patients reported product-related adverse events.

### 3.4. Dietary Intake

Table 3 presents the dietary intake of patients at various timepoints. Dietary intake was comparable across the groups at baseline. Preoperatively, Group SS and SS-E who received supplementation showed significantly higher intakes of energy, protein, carbohydrate, and fat than Group DS (*p* < 0.001). At 30- and 90-days post-discharge, Group SS-E reported significantly higher intakes of energy, protein, carbohydrate, and fat than Group SS and DS (*p* < 0.001).

### 3.5. Study Outcomes

**Figure 2:**
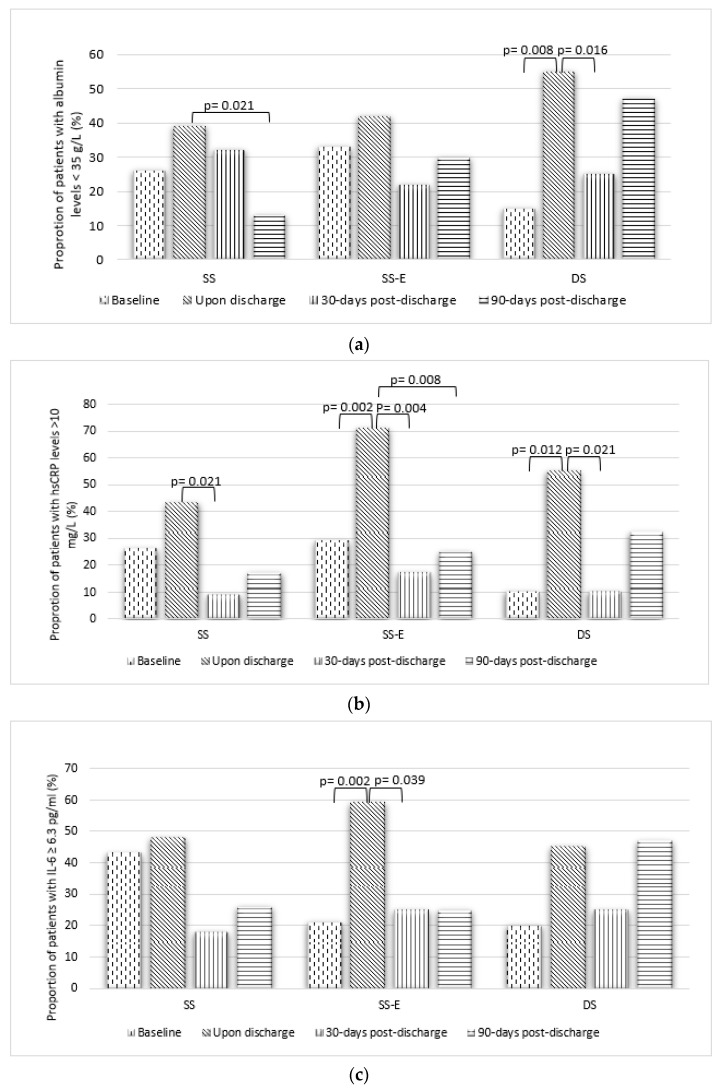
Changes in the proportion of patients with (**a**) albumin < 35 g/L, (**b**) hsCRP > 10 mg/L and (**c**) IL-6 ≥ 6.3 pg/mL. *p* value indicated significance within groups between timepoints determined by McNemar test.

An intent-to-treat analysis of the primary and secondary outcomes is presented in Table 4. The changes in the proportion of patients with inflammation-based prognostic markers above the cut-off values across the timepoints are shown in Figure 2 on a per-protocol basis.

#### 3.5.1. Primary and Secondary Outcomes

Repeated measures analysis showed no differences in changes over time between the groups (*p* > 0.050), as shown in Table 4. However, after preoperative supplementation, there was a significant difference in mean weight and BMI preoperatively between Group SS and Group DS after adjusting for baseline values. The weight of Group SS and Group DS was 66.1 ± 15.3 and 62.5 ± 12.0 kg, respectively (F (2,86) = 4.870, *p* = 0.010). The BMI of Group SS and Group DS was 26.8 ± 6.8 and 26.1 ± 6.7 kg/m^2^, respectively (F (2,86) = 4.010, *p* = 0.022).

At 90-days post-discharge, extended supplementation resulted in significant differences in mean BMI and handgrip strength between Group SS-E and Group DS after adjusting for baseline values. The group receiving the extended supplementation recorded significantly higher values for both these variables when compared to the group that received dietary counselling. The BMI of Group SS-E and Group DS was 27.2 ± 6.5 and 24.4 ± 4.7 kg/m^2^, respectively (F (2,70) = 3.228, *p* = 0.046). The handgrip strength of Group SS-E and Group DS was 26 ± 9 and 24 ± 6 kgF, respectively (F (2,68) = 3.260, *p* = 0.044).

The serum albumin, hsCRP and IL-6 levels categorised based on cut-off values and the within-group changes in proportions of patients are presented in Figure 2. There was a significant increase in the proportion of patients with low serum albumin < 35 g/L in Group DS between baseline and upon discharge (15% to 55%, *p* = 0.008), whereas there were no significant changes in albumin status in Groups SS and SS-E who were supplemented preoperatively. Nevertheless, at 30-days post-discharge, the proportion of patients in Group DS with low serum albumin < 35 g/L had dropped significantly from upon discharge (55% to 25%, *p* = 0.016). At 90-days post-discharge, Group SS had significantly lower patients with albumin < 35 g/L as compared with upon discharge (39% to 13%, *p* = 0.021).

There were significant increases in the proportions of patients with high hsCRP > 10 mg/L in Group SS-E (29% to 71%, *p* = 0.002) and Group DS (10% to 55%, *p* = 0.012) between baseline and upon discharge. However, the proportions of patients with high hsCRP > 10 mg/L reduced significantly in all groups at 30-days post-discharge as compared with upon discharge. At 90-days post-discharge, Group SS-E had significantly lower proportion of patients with hsCRP > 10 mg/L as compared with upon discharge (71% to 25%, *p* = 0.008).

Group SS-E had significant increase in the proportions of patients with high IL-6 ≥ 6.3 pg/mL between baseline and upon discharge (21% to 59%, *p* = 0.002). However, the proportion of patients with high IL-6 ≥ 6.3 pg/mL reduced significantly at 30-days post-discharge compared with upon discharge (59% to 25 %, *p* = 0.039). Similar trends were noted in Group SS and Group DS for IL-6, although they were not statistically significant.

#### 3.5.2. Inflammatory Prognostic Markers

**Figure 3:**
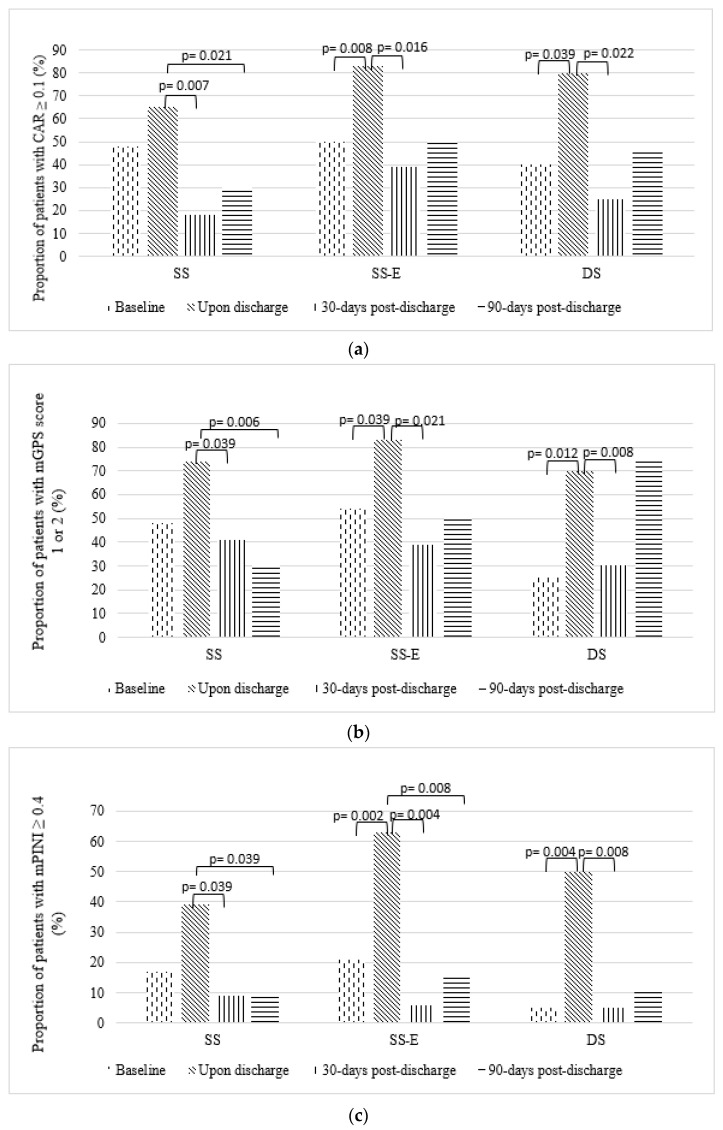

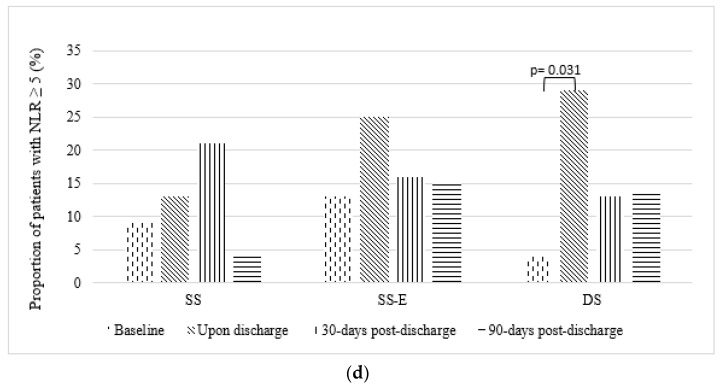
Changes in the proportion of patients with (**a**) CAR ≥ 0.1, (**b**) mGPS score 1 or 2, (**c**) mPINI ≥ 0.4 and (**d**) NLR ≥ 5. *p* value indicated significance within groups between timepoints determined by McNemar test.

Figure 3 shows the categorical distribution of inflammatory prognostic markers by group, at baseline, upon discharge, 30- and 90-days post-discharge. There was a significant increase in the proportion of patients with poor prognosis based on CAR ≥ 0.1 in Group SS-E (50% to 83%, *p* = 0.008) and Group DS (40% to 80%, *p* = 0.039) between baseline and upon discharge. However, the proportions of patients with CAR ≥ 0.1 were significantly lowered in all groups at 30-days post-discharge as compared with upon discharge. At 90-days post-discharge, the proportion of patients with CAR ≥ 0.1 significantly decreased in Group SS as compared with upon discharge (65% to 30%, *p* = 0.021).

A similar trend was observed for mPINI. The proportion of patients with mPINI ≥ 0.4 indicating poor prognosis between baseline and upon discharge was significantly increased in Groups SS-E (21% to 63%, *p* = 0.002) and Group DS (5% to 50%, *p* = 0.004). However, at 30-days post-discharge, the proportion of patients with mPINI ≥ 0.4 was significantly lowered in all groups as compared with upon discharge. At 90-days post-discharge, there was a significant drop in patients with mPINI ≥ 0.4 in Group SS (39% to 9%, *p* = 0.039) and Group SS-E (63% to 15%, *p* = 0.008) compared with upon discharge.

For mGPS, the proportion of patients with poor prognosis score 1 or 2 was significantly higher in Group SS-E (54% to 83%, *p* = 0.039) and Group DS (25% to 70%, *p* = 0.012) between baseline and upon discharge. The proportions of patients with mGPS score 1 or 2 were significantly lower in all groups at 30-days post-discharge as compared with upon discharge. At 90 days post-discharge, the proportion of patients with mGPS score 1 or 2 was significantly lower than upon discharge only in Group SS (74% to 30%, *p* = 0.006).

For NLR, there was a significant increase in patients with high NLR between baseline and upon discharge for Group DS (4% to 29%, *p* = 0.031). No other significant shifts in the proportion of patients were observed for other groups over the timepoints.

## 4. Discussion

Our study is among the few studies in Asia reporting the effects of perioperative ONS supplementation among well to mild–moderately malnourished breast and colorectal cancer patients. The results showed that preoperative supplementation in these patients assists in maintaining weight and BMI prior to surgery while postoperative supplementation seems to confer modest benefits in improving handgrip strength and BMI as well as lowering the proportion of patients with elevated hsCRP and inflammatory prognostic marker, mPINI, at 90-days post-discharge.

Our study showed beneficial effects of preoperative supplementation with ONS ranging from 6 to 14 days on maintaining weight and BMI prior to surgery. At baseline, the majority of our breast and colorectal cancer patients were overweight or obese and this finding was similar to other studies [7,8], as obesity was a major risk factor of cancer that positively correlated with cancer incidence, aggressiveness, and mortality [25]. However, BMI may be insufficient to characterise the nutrition status in cancer patients. The AND/ASPEN nutrition assessment tool revealed that one-third of the patients were mild-to-moderately malnourished as it assessed factors beyond BMI including reduced dietary intake, muscle loss and weight loss [26]. The non-supplemented group reported significantly lower dietary intake as compared to the supplemented groups prior to surgery. It has been reported that dietary intake, in terms of energy and protein intake, had a highly significant positive correlation with weight change among cancer patients [27]. Compared to dietary advice with usual diet alone, oral nutrition supplements were effective in attenuating weight loss and improving dietary intake with little suppression on food intake [18]. Besides that, prolonged significant weight loss (>5% or BMI reduction or category change) after diagnosis was associated with reduced long-term survival in cancer [28]. In order to circumvent the catabolic stress induced by the surgery, the European Society of Parenteral and Enteral Nutrition (ESPEN) guidelines for perioperative nutrition (2020) recommended the use of preoperative oral nutrition supplements irrespective of the nutritional status as the clinical benefits outweigh the surgical risks [29]. A latency of up to 12 weeks from the time of diagnosis to curative surgery has shown to have no impact on overall survival. Hence, a short delay in surgery to provide preoperative nutrition intervention for patients with nutrition risk could pose greater benefits to the patients [30]. The beneficial effects reported by this study is in line with the results of a review that included studies with preoperative supplementation ranging from 5 days to 4 weeks [6] and adds further strength to the evidence to recommend pre-operative nutrition intervention.

At present, the evidence for postoperative supplementation remains inconclusive. A systematic review revealed high heterogeneity in the evidence of the effectiveness of postoperative oral nutrition supplementation [17]. Studies have reported on the improvement of nutritional status with postoperative feeding during the acute phase up to 7 days after surgery [31,32], the first 60-days after surgery [33], and the first 120-days after surgery [34]. The current study demonstrated that among patients undergoing elective surgery for breast and colorectal cancer with mild-to-moderate malnutrition, a prolonged supplementation until 90 days post-discharge significantly improved handgrip strength. Handgrip strength has been positively correlated with overall muscle strength, functional ability, and nutrition status in several chronic diseases including advanced cancer [35]. It has also been shown by other studies to correlate with reduced morbidity after cancer surgery [36] and overall improvement of quality of life among cancer patients [37,38]. A recent study by Tan et al. (2021) reported that 3-month post-discharge supplementation among colorectal cancer patients with nutrition risk resulted in higher skeletal muscle index and lower sarcopenia prevalence as compared to those receiving dietary advice alone [39]. During the disease trajectory, muscle protein is broken down and muscle synthesis diminished to redirect the protein source for the synthesis of pro-inflammatory cytokines and acute-phase proteins [9,40]. Hence, muscle depletion could co-exist with obesity in the absence of significant weight change [41]. Patients with sarcopenia or significant muscle loss had a 35% increased risk of postoperative complications across gastrointestinal cancers [42]. Nutrition exerts an anabolic effect on muscle protein after the tumour was resected [40] and this could have potential benefits in improving recovery post-surgery.

In the present study, perioperative supplementation with ONS showed no remarkable findings on the inflammatory markers and inflammatory-based prognostic markers. In the current study, the proportions of patients with elevated inflammatory markers in all groups increased at discharge in response to the surgery but reduced at 30-days post-discharge. Surgery exerts stress on patients because it is an invasive approach that induces direct injury by causing wound as well as indirect injury through driving immunologic, metabolic, and hormonal derangements in the body [16]. Surgical stress renders the body in a catabolic state through the release of stress hormones and inflammatory cytokines and brings about complications [9,16,43]. The alteration is self-terminated upon completion of reparatory activity [44] or prolonged to further damage the host tissue in the case of systemic inflammatory response syndrome (SIRS) or sepsis [45].

Inflammation-based prognostic markers have been extensively studied among various cancer types based on the premise that these patients experience chronic inflammation from the primary tumour and treatment modalities [13]. These markers also indicate the balance between the inflammatory state and nutritional status of the patients [13,15,46,47]. The evidence of ONS on these inflammatory prognostic markers remains scarce. An earlier study among oral cavity cancer patients found that pre-treatment supplementation decreased CAR, but the reduction was not statistically significant [48]. Albumin is the most abundant source of protein in the body and its turnover is affected by the disease states and dietary intake [49]. It is a useful prognostic marker in various types of cancer and hypoalbuminemia was associated strongly with poor cancer survival, higher postoperative complications, and longer length of hospital stay [50,51]. In the present study, the proportion of patients with albumin level < 35 g/L significantly increased at discharge in Group DS and reduced at 30-days post-discharge, though the changes in albumin were not significant between groups. Furthermore, the proportions of patients with elevated inflammation-based prognostic markers in all groups increased at discharge in response to the surgery but reduced at 30-days post-discharge. At 90-days post-discharge, the proportion of patients with elevated hsCRP significantly reduced in Group SS-E when compared to upon discharge. Elevated hsCRP is associated with increased risk of cancer-related mortality and individuals with the highest hsCRP levels have a 25% greater risk of total cancer mortality as evidenced by a recent meta-analysis [52].

Similarly, at 90-days post-discharge, the proportion of patients with mPINI ≥ 0.4 significantly reduced in Group SS-E when compared to upon discharge. The expression of mPINI was based on CAR values and a cut off value of 0.4 was positively associated with weight loss and malnutrition in patients with gastrointestinal tumours [12]. At this juncture, it is noteworthy that the choice of clinical prognostic marker use and respective cut-off values are specific to cancer types and the treatment modalities due to the variety of systemic inflammation status [53]. Also, these markers have shown to be more accurate in predicting overall survival and recurrence-free survival among colorectal cancer patients when measured postoperatively [54]. Thus, the role of ONS in improving inflammation-based prognostic markers requires further study.

The role of baseline nutrition status of the patients in determining the benefit of perioperative nutrition support needs further evaluation. Recent ESPEN guideline on perioperative nutrition (2020) suggested the use of preoperative supplementation irrespective of the nutrition status and reported beneficial effects on postoperative feeding [29]. Earlier, Kabata et al. (2015) also found less postoperative complications and better blood parameters among well-nourished cancer patients [5]. Other guidelines concluded that patients should then be evaluated for nutrition risk preoperatively [55] and ONS should be reserved for those cancer patients who are moderately or severely malnourished with an expectation of not being able to achieve nutrition requirements within 7–14 days postoperatively [56]. The findings from the present study showed modest benefits of the use of ONS in non-severely malnourished cancer patients, with significant benefits restricted to handgrip strength and body weight maintenance. Miller et al. (2013) explained that the identification of patients who will benefit from nutrition support is based on the patient’s current nutrition status, the severity of the surgical insult, and the possibility of anatomic alterations that predispose the patient to nutrition risk [55]. In the present study, majority of the patients was well or mild-to-moderately malnourished, undergoing elective surgery instead of emergency surgery with early-staged cancers, and having low risk of anatomic alterations from the breast and colorectal cancer surgeries. This could explain the modest results of the present study.

Despite adding to the limited evidence on the use of ONS in cancer patients with mild–moderate malnutrition undergoing elective surgery, specifically in an upper–middle income scenario, the authors acknowledge its limitations. Given that the patients in this trial were not severely malnourished, the effects of the intervention could have been attenuated. Therefore, this study may not have been sufficiently powered to detect the small effects in the secondary outcomes such as inflammatory and prognostic markers. This study did not stratify patients in terms of cancer type in data analyses. However, existing literature shows that the systemic response to surgery among several cancers including breast and colorectal cancers is similar [57] and the proportion of breast and colorectal cancers were comparable between the groups. Future studies could further explore the perioperative use of ONS in larger studies that could allow better control for cancer type, age and surgical variables.

## 5. Conclusions

This study demonstrated that perioperative supplementation has modest benefits in attenuating weight loss preoperatively while improving handgrip strength and inflammatory prognostic markers postoperatively with an extended supplementation up to 90-days post-discharge in well-nourished and mild-to-moderately malnourished breast and colorectal cancer patients undergoing elective surgery.

## Figures and Tables

**Table 1:** Baseline characteristics of patients.

Variable	Group SS *N* = 30	Group SS-E *N* = 31	Group DS *N* = 30	*p*-Value
Age (year), median (Q1–Q3)	60 (50–65)	60 (45–67)	61 (46–65)	0.050
Sex				0.855
Male	6 (20)	10 (32)	6 (23)	-
Female	24 (80)	21 (68)	24 (77)	-
Cancer types				0.357
Breast cancer	21 (68)	15 (48)	17 (57)	-
Colorectal cancer	9 (32)	16 (52)	13 (43)	-
Comorbidities				
Diabetes mellitus	9 (30)	6 (19)	9 (30)	0.551
Hypertension	14 (47)	14 (45)	13 (43)	0.967
Cardiovascular diseases	10 (33)	6 (19)	9 (30)	0.441
Body weight (kg), mean ± SD	65.6 ± 2.8	65.0 ± 2.3	62.8 ± 2.2	0.714
Body Mass Index (kg/m^2^), mean ± SD	26.6 ± 6.8	26.1 ± 5.0	26.3 ± 7.0	0.839
Underweight, <18.5	3 (10)	3 (10)	2 (7)	-
Normal, 18.5–24.9	12 (40)	10 (32)	14 (47)	-
Overweight, ≥25.0	5 (17)	10 (32)	7 (23)	-
Obese, ≥30.0	10 (33)	8 (26)	7 (23)	-
Nutritional status, AND/ASPEN				0.649
Well-nourished	10 (33)	9 (29)	9 (30)	-
Mild-to-moderately malnourished	8 (27)	14 (45)	11 (37)	-
Severely malnourished	12 (40)	8 (26)	10 (33)	-
Handgrip strength (kgF), mean ± SD	27 ± 8	27 ± 8	25 ± 6	0.787
Albumin (g/L), mean ± SD	40 ± 1	38 ± 2	41 ± 1	0.323
hsCRP (mg/L), mean ± SD	7.7 ± 2.6	11.1 ± 3.2	5.8 ± 2.0	0.401
IL-6 (pg/mL), mean ± SD	7.7 ± 2.7	7.1 ± 2.0	4.5 ± 0.6	0.535

All values were expressed as *n* (%) unless otherwise stated. *p* values were calculated using ANOVA for normally distributed data, Kruskal Wallis test for skewed data, and Chi-Square test for categorical data.

**Table 2:** Supplementation duration and compliance of patients.

Variable	Group SS *N* = 26	Group SS-E *N* = 21	Group DS *N* = 25	*p*-Value
Supplementation Duration, days, median (Q1–Q3)				
Preoperative	8 (6–14)	7 (4–13)	-	0.144
30-days post-discharge	-	29 (26–35)	-	-
90-days post-discharge	-	63 (56–68)	-	-
30&90-days post-discharge	-	97 (85–103)	-	-
Compliance to ONS, %, mean ± SD				
Preoperative	84 ± 19	92 ± 17	-	0.154
30-days post-discharge	-	77 ± 20	-	-
90-days post-discharge	-	75 ± 20	-	-
30&90-days post-discharge	-	75 ± 16	-	-

*p* values were based on Mann–Whitney U tests for between-group difference for skewed data and independent *t* tests for normally distributed data.

**Table 3:** Dietary intake of patients.

Variables	Group SS *N* = 26	Group SS-E *N* = 21	Group DS *N* = 25	*p*-Value
Energy (kcal/d)				
Baseline	1134 ± 394	1179 ± 317	1148 ± 342	0.910
Preoperative	1923 ± 470 ^a^	1885 ± 255 ^a^	1115 ± 353 ^b^	<0.001
30-days post-discharge	1254 ± 462 ^a^	1781 ± 307 ^b^	1238 ± 314 ^a^	<0.001
90-days post-discharge	1287 ± 373 ^a^	1963 ± 326 ^b^	1346 ± 318 ^a^	<0.001
Protein (g/d)				
Baseline	46 ± 20	47 ± 16	45 ± 17	0.934
Preoperative	79 ± 21 ^a^	81 ± 16 ^a^	45 ± 16 ^b^	<0.001
30-days post-discharge	51 ± 21 ^a^	79 ± 17 ^b^	50 ± 13 ^a^	<0.001
90-days post-discharge	52 ± 20 ^a^	85 ± 18 ^b^	52 ± 13 ^a^	<0.001
Carbohydrate (g/d)				
Baseline	154 ± 55	150 ± 42	146 ± 48	0.838
Preoperative	227 ± 54 ^a^	221 ± 37 ^a^	147 ± 50 ^b^	<0.001
30-days post-discharge	166 ± 67 ^a^	211 ± 41 ^b^	154 ± 43 ^a^	0.001
90-days post-discharge	164 ± 51 ^a^	232 ± 46 ^b^	177 ± 46 ^a^	<0.001
Fat (g/d)				
Baseline	36 ± 16	43 ± 17	43 ± 14	0.233
Preoperative	74 ± 23 ^a^	73 ± 12 ^a^	39 ± 13 ^b^	<0.001
30-days post-discharge	41 ± 17 ^a^	67 ± 15 ^b^	45 ± 16 ^a^	<0.001
90-days post-discharge	46 ± 15 ^a^	77 ± 16 ^b^	48 ± 15 ^a^	<0.001

All values were expressed as mean ± standard deviation. *p* values were based on ANOVA for between-group differences. Groups with different alphabetical superscripts were significantly different. Bonferroni test was employed for post hoc analyses.

**Table 4:** Evaluation of primary and secondary outcomes between groups.

Variables	Group SS	Group SS-E	Group DS	Time * Group
	*N* = 30	*N* = 31	*N* = 30	*p*-Value
Weight (kg)				0.214
Baseline	65.6 ± 15.5	65.0 ± 13.2	62.8 ± 12.5	
Preoperative	66.1 ± 15.3 ^a^	65.2 ± 12.9 ^a,b^	62.5 ± 12.0 ^b^	
30-days post-discharge	65.3 ± 15.4	64.5 ± 12.8	61.3 ± 11.9	
90-days post-discharge	65.9 ± 15.3	65.5 ± 13.5	61.7 ± 12.2	
Body mass index (kg/m^2^)				0.172
Baseline	26.6 ± 6.8	26.2 ± 5.0	26.2 ± 6.9	
Preoperative	26.8 ± 6.8 ^a^	26.3 ± 5.0 ^a,b^	26.1 ± 6.7 ^b^	
30-days post-discharge	26.5 ± 6.7	26.1 ± 4.9	25.6 ± 6.7	
90-days post-discharge	26.7 ± 6.6 ^a^	26.5 ± 5.2 ^a^	25.8 ± 6.6 ^b^	
Albumin (g/L)				0.633
Baseline	39 ± 8	38 ± 8	41 ± 7	
Preoperative	38 ± 7	38 ± 8	38 ± 9	
30-days post-discharge	38 ± 8	39 ± 5	39 ± 9	
90-days post-discharge	39 ± 6	39 ± 8	38 ± 7	
Handgrip strength (kgF)				0.096
Baseline	27 ± 8	27 ± 8	25 ± 6	
Preoperative	26 ± 8	25 ± 8	25 ± 7	
30-days post-discharge	27 ± 8	27 ± 6	25 ± 7	
90-days post-discharge	27 ± 9 ^a,b^	28 ± 7 ^a^	25 ± 6 ^b^	
hsCRP (mg/L)				0.525
Baseline	7.5 ± 13.1	10.8 ± 17.2	5.5 ± 9.8	
Preoperative	6.5 ± 7.4	10.9 ± 16.4	5.5 ± 8.6	
30-days post-discharge	7.5 ± 21.0	8.2 ± 17.4	3.7 ± 4.1	
90-days post-discharge	4.7 ± 5.9	14.1 ± 36.0	14.4 ± 38.0	
IL-6 (pg/mL)				0.223
Baseline	7.4 ± 13.6	6.8 ± 10.6	4.3 ± 2.8	
Preoperative	13.6 ± 29.8	8.0 ± 12.9	5.9 ± 4.5	
30-days post-discharge	7.6 ± 15.1	5.1 ± 2.5	4.7 ± 3.1	
90-days post-discharge	4.7 ± 2.6	6.1 ± 6.2	9.3 ± 18.3	

All data expressed as mean ± SD. GLM Repeated Measure was used to detect time * group interactions. Groups with different alphabetical superscripts were significantly different using ANCOVA when adjusted for baseline values. Bonferroni test was employed for post hoc analyses.

## Data Availability

Not applicable.
